# Supplementary material for: Bile acid-dependent transcription factors and chromatin accessibility determine regional heterogeneity of intestinal antimicrobial peptides
Source: Nat Commun. 2023 Aug 22;14:5093. doi: 10.1038/s41467-023-40565-7 (PMC10444805; doi:10.1038/s41467-023-40565-7)
Supplement: Supplementary file 1 — Supplementary Information [file 41467_2023_40565_MOESM1_ESM.pdf]

a

Human epithelial cell cluster names	Representative genes		Tissue enrichment (Postnatal)						Enrichment (Prenatal)		
	Functional genes	Transcription factors (RSS top4)	D	J	I	A	C	R	Pro. SI	Dis. SI	LI
E01-Stem cells	*LGR5, RGMB, SMOC2, ASCL2*	*BCL11A, ZNF121, NFIX, TAL1*	+	+	++	+	+	+	++	++	+
E02-TA	*MCM5, TOP2A, CCNA2, PCLAF*	*HES6, HMGA2, E2F8, TP53*	+	+	+/-	++	+	+	++	++	++
E03-Immature enterocytes	High expression of: *OLFM4, DMBT1, CBR1, AKR7A3*<br>Low expression of: functional genes in well differentiated enterosytes	*DBP, MAF, HNF1A, NR1I2*	+++	+++	++	+++	+++	+++	+++	++	+++
E04-*APOA1*+ Enterocytes	*ALDOB, FABP2, ANPEP, ADIRF*	*MAF, NR1I3, DBP, PRDM16*	+++	+++	+++	-	-	-	++	+++	++
E05-*CA1*+Enterocytes	*CA1, AQP8, PEBP1, FABP5*	*EPAS1, TFAP4, TFCP2L1, HES2*	-	-	+/-	++	++	++	-	+/-	++
E06-*BEST4*+ Enterocytes	*CA7, BEST4, SPIB, CA4*	*HES4, MEIS1, RBPJ, TFAP2E*	+	+	+	+/-	+	+	+	+	+
E07-PI3+ Enterocytes	*LCN2, PI3, S100A11, S100A6*	*PRRX2, HES2, ZNF257, RARG*	-	-	-	+/-	+/-	+/-	-	-	+/-
E08-*IGKC*+ Enterocytes	*IGHA1, JCHAIN, IGKC, HSP90AA1*	*TCF21, PURA, IRX3, VAX2*	-	-	-	+	+	+	-	-	+
E09-Microfold cells	*CCL20, CCL23, MIA, PTMA*	*ZXDA, ARNT2, NFE2L3, HOXD13*	-	-	+/-	+	+/-	+	-	+/-	+/-
E10-Enteroendocrine cells	*CHGA, PCSK1N, SCGN, CHGB*	*PAX6, ISL1, ASCL1, ARX*	+/-	+	+	+	+	+	+	+	+
E11-Tuft cells	*SH2D6, IRAG2, AZGP1, FYB1*	*ZFHX3, RUNX1, HOXA3, HOXA4*	+/-	+	+/-	+/-	+	+/-	+/-	+/-	+/-
E12-Goblet cells	*TFF3, MUC2, FCGBP, ITLN1*	*ATOH1, TBX10, ARNT, FOXP4*	+	+	+	+	++	+	+	+	+
E13-Paneth cells	*DEFA5, DEFA6, REG3A, REG1A*	*FOXL1, FOXF2, TCF21, NKX2-3*	+/-	+/-	+	-	+/-	+/-	+/-	+	-
E14-*PGC*+mucous cells	*LYZ, ANXA10, TFF2, PGC*	*GSC, FOXQ1, ZSCAN4, MECOM*	+	+/-	+/-	-	-	-	+/-	+/-	-

b

HIO epithelial cell cluster names	Representative genes	
	Functional genes	Transcription factors (RSS top4)
HIO-01-Stem cells	*LGR5, RGMB, HMGCS2, COL2A1*	*HOXC8, HOXD9, HMGA2, NFYC*
HIO-02-Immature enterocytes	*OLFM4, DMBT1, CBR1, AKR7A3*	*HNF1A, MAF, GATA5, DBP*
HIO-03-Enterocytes	*FABP1, APOB, SERPINA1, RBP2*	*NR1D1, NR1I3, DBP, MAF*
HIO-04-*BEST4*+ Enterocytes	*FKBP1A, SPIB, BEST4, CA7*	*TCF3, RBPJ, NFE2L3, HES4*
HIO-05-Enteroendocrine cells	*CADPS, CPE, CHGA, CHGB*	*ASCL1, PAX3, PAX6, NHLH1*
HIO-06-Goblet cells	*SELENOM, ST6GALNAC1,TFF3, HPCAL1*	*BCL6B, FOXD4L5, ARNT, FOXD4L1*
HIO-07-*PGC*+mucous cells	*LYZ, SPINK1, PGC, VSIG2*	*FOXQ1, ZSCAN4, RBPJ, AHR*
HIO-08-Basal like cells	*ANXA1, S100A11, HSPB1, MDK*	*RFX8, TP73, KLF14, LHX2*
HIO-09-Ciliated like cells	*ZMYND10, CFAP126, TPPP3, PIFO*	*RFX8, TP73, KLF14, LHX2*

c

Mouse epithelial cell cluster names	Representative genes		Tissue enrichment	
	Functional genes	Transcription factors (RSS top4)	Ileum	Colon
E01-Stem cells	*Kcnq1, Sema5a, Cftr, Camk1d*	*Hmga2, Mybl2, Esrrg, Prdm5*	++	++
E02-TA	*Top2a, Cenpp, Casc5, Mki67*	*Pou2f1, Nup133, Mybl2, Mybl11*	++	++
E03-Immature enterocytes	Ileum: *St3gal4, Malrd1, Sis, Pcsk5*<br>Colon: *Mecom, Satb2, Car2, Htr4*	Ileum: *Maf, Mtf1, Barx2, Nr1h3*<br>Colon: *Hoxa9, Hnf1b, Mecom, Grhl2*	++	++
E04-*Apoa1*+ Enterocytes	*Apoa1, St3gal4, Slc5a1, Ace2*	*Maf, Barx2, Mtf1, Tbx3*	++	-
E05-*Aqp8*+ Colonocytes	*Cyp2c55, Lypd8, Slc26a3,Aqp8*	*Hif1a, Foxd2, Hoxb13, Atf3*	-	++
E06-*Fcgbp*+ Colonocytes	*Lypd8, S100a6, Krt8, Fth1*	*Hoxa13, Hoxb13, Cdx1, Hif1a*	-	+
E07-Microfold cells	*Esrrg, Dapk2, Pard3b, Akt3*	*Rarb, Sox8, Hoxd10, Onecut2*	+/-	+/-
E08-Enteroendocrine cells	*Kcnb2, Rimbp2, Cacna1a, Ptprn2*	*Isl1, Arx, Slc18a1, Crebl2*	+	+
E09-Tuft cells	*Dclk1, Sh2d6, Dgki, Nebl*	*Nfatc1, Runx1, Gm14434, Hsf2*	+/-	+
E10-Immature goblet cells (Colon)	*9030622O22Rik, Kcnma1, Slc12a8, Oit1*	*Etv5, Nfatc2, Hoxd1, Mitf*		++
E11-Goblet cells	*Muc2, Fcgbp, Tff3, Bcas1*	*Tff3, Mxd4, Tbx3,Tead4*	+	+
E12-Paneth cells	*Mmp7, Lyz1, Defa24, Defa3*	*Klf15, Bhlha15, Bcl6, Mef2c*	++	-

Supplemental Fig. 1 | Summary of epithelial cell subsets in human and mouse intestine.
a Overview of epithelial cell cluster characteristics from pre- and postnatal human intestine.
b Overview of epithelial cell cluster characteristics from human intestinal organoids.
c Overview of epithelial cell cluster characteristics from mouse intestine. The subset-specific TFs were obtained by regulon specificity score (RSS) and functional genes were obtained by differential expression analysis.
+++ indicates that the cell subset is detected and the proportion of it in the epithelial compartment of the respective region is more than 30%, ++ indicates that is more than 10% but less than 30%, + indicates that is more than 1% but less than 10%, -/+ indicates that is less than 1%, and - indicates no detection of this cell subset.

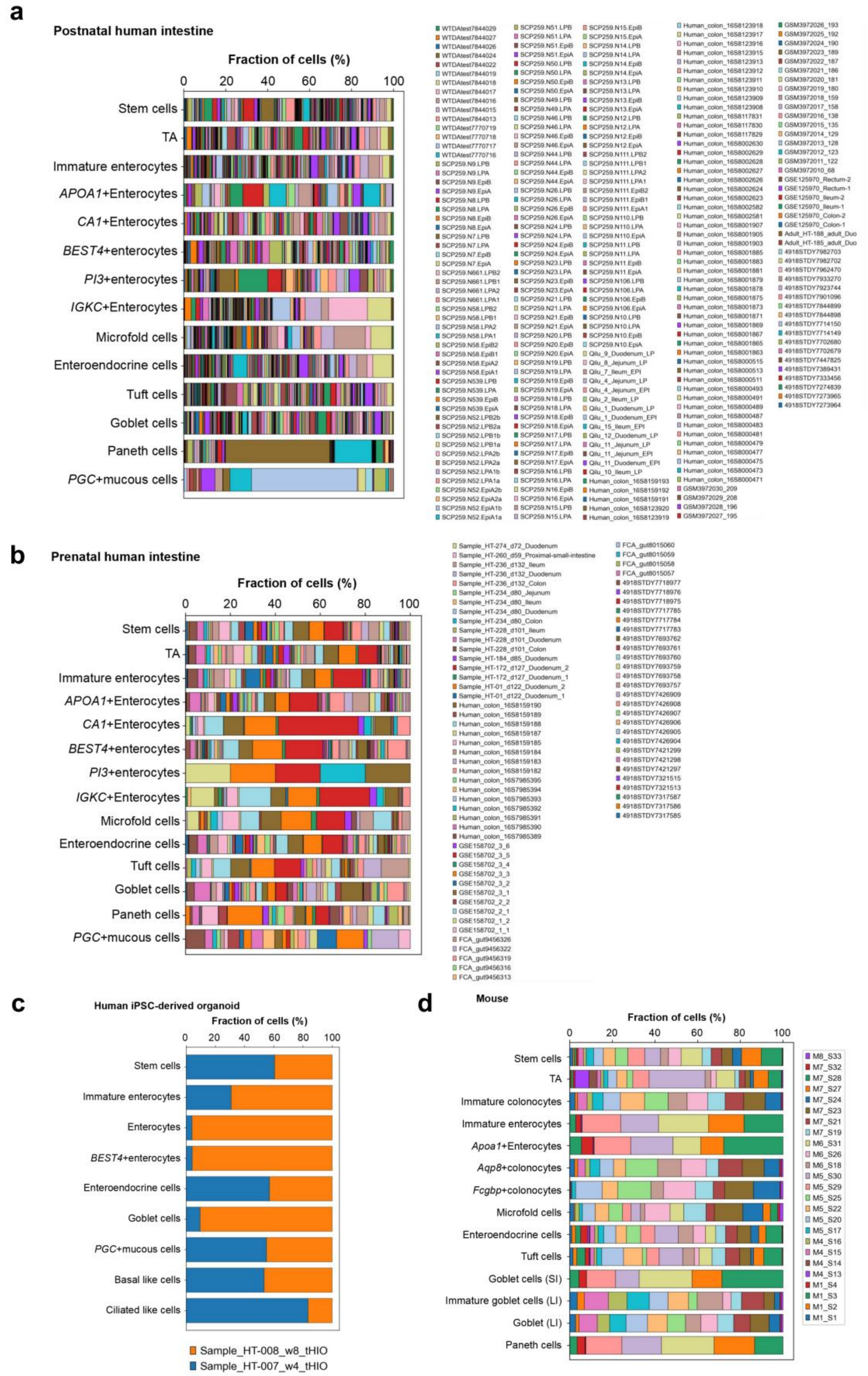

Supplemental Fig. 2 | The distribution of samples in various cell clusters.

a-d Bar plots showing the contributions of different samples in various cell clusters in postnatal human intestine(a), prenatal human intestine (b), human iPSC-derived organoid (c) and mouse (d).

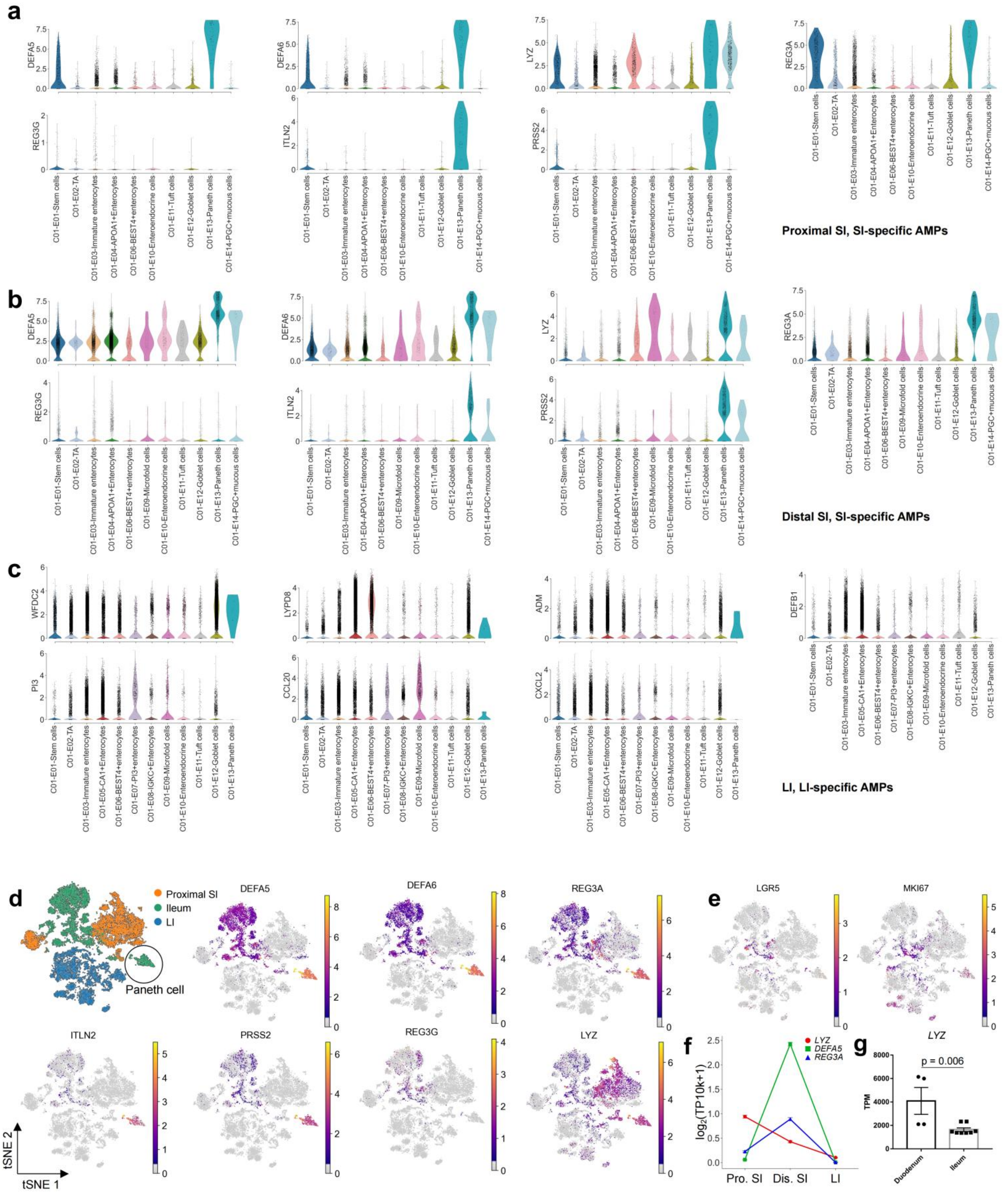
a
DEFA5
DEFA6
LYZ
REG3A
REG3G
ITLN2
PRSS2
C01-E01-Stem cells
C01-E02-TA
C01-E03-Immature enterocytes
C01-E04-APOA1+Enterocytes
C01-E06-BEST4+enterocytes
C01-E10-Enteroendocrine cells
C01-E11-Tuft cells
C01-E12-Goblet cells
C01-E13-Paneth cells
C01-E14-PGC+mucous cells
Proximal SI, SI-specific AMPs
b
C01-E09-Microfold cells
Distal SI, SI-specific AMPs
c
WFDC2
LYPD8
ADM
DEFB1
PI3
CCL20
CXCL2
C01-E05-CA1+Enterocytes
C01-E07-PI3+enterocytes
C01-E08-IGKC+Enterocytes
LI, LI-specific AMPs
d
Proximal SI
Ileum
LI
Paneth cell
tSNE 2
tSNE 1
e
LGR5
MKI67
f
log2(TP10k+1)
Pro. SI
Dis. SI
LI
g
p = 0.006
TPM
Duodenum
Ileum

Supplemental Fig. 3 | Regional heterogeneity and cell type specificity of AMPs in intestinal epithelial cells.

a-c Violin plots showing the expression of SI-specific AMPs in the proximal (a) and distal SI (b), and LI-specific AMPs in the LI (c).

d t-SNE embedding showing the expression of SI-specific AMPs (*DEFA5*, *DEFA6*, *REG3A*, *REG3G*, *LYZ*, *ITLN2*, *PRSS2*, and *REG3G*) in epithelial cells. The same number of cells (10,000 cells) were randomly sampled from the proximal SI, ileum, and LI.

e t-SNE embedding showing the expression of stem cell (*LGR5*) and TA (*MKI67*) markers in epithelial cells. The same number of cells (10,000 cells) were randomly sampled from the proximal SI, ileum, and LI.

f Line chart showing the expression of three SI-specific AMPs (*LYZ, DEFA5, REG3A*) in proximal SI (Pro. SI), distal SI (Dis. SI) and LI in scRNA seq data. The length of the error bars is a 95% confidence interval for the mean.

g Bar plot of bulk RNA-seq data (E-MTAB-1733) showing that *LYZ* has a significantly higher expression level in the duodenum (n = 4 biologically independent samples) than in the ileum (n = 8 biologically independent samples). Data are presented as mean ± SEM. All *p*-values were calculated and reported using two-tailed Student's *t*-test. Source data are provided as a Source Data file.

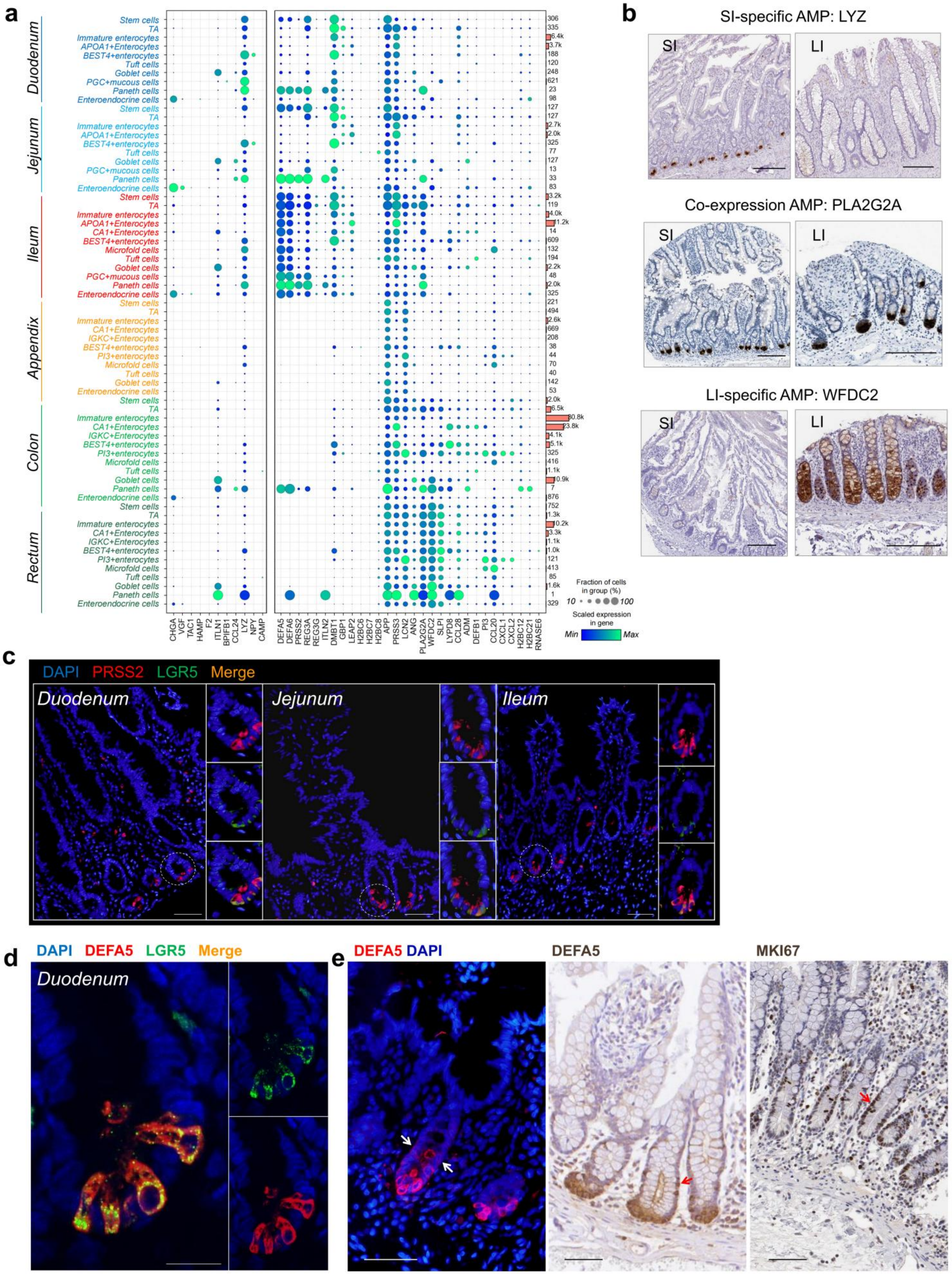

a
Duodenum
Jejunum
Ileum
Appendix
Colon
Rectum
Stem cells
TA
Immature enterocytes
APOA1+Enterocytes
BEST4+enterocytes
Tuft cells
Goblet cells
PGC+mucous cells
Paneth cells
Enteroendocrine cells
CA1+Enterocytes
Microfold cells
IGKC+Enterocytes
PI3+enterocytes
CHGA VGF TAC1 HAMP F2 ITLN1 BPIFB1 CCL24 LYZ NPY CAMP
DEFA5 DEFA6 PRSS2 REG3A REG3G ITLN2 DMBT1 GBP1 LEAP2 H2BC6 H2BC7 H2BC8 APP PRSS3 LCN2 ANG PLA2G2A WFDC2 SLPI LYPD8 CCL28 ADM DEFB1 PI3 CCL20 CXCL1 CXCL2 H2BC12 H2BC21 RNASE6
Fraction of cells in group (%)
10
100
Scaled expression in gene
Min
Max
b
SI-specific AMP: LYZ
Co-expression AMP: PLA2G2A
LI-specific AMP: WFDC2
SI
LI
c
DAPI PRSS2 LGR5 Merge
Duodenum
Jejunum
Ileum
d
DAPI DEFA5 LGR5 Merge
Duodenum
e
DEFA5 DAPI
DEFA5
MKI67

Supplemental Fig. 4 | Multi-omics verification of the regional heterogeneity of antimicrobial peptides expressed on the crypt-villus axis and different intestinal regions.

a Dot plot showing the diversity of AMPs expression pattern in different intestinal regions and cell specificity of AMPs.

b Bowel sections from human intestine are immunostained for LYZ, PLA2G2A and WFDC2, from https://proteinatlas.org. Scale bars, 200µm.

c Bowel sections from intestine are immunofluorescent stained for PRSS2 (red), LGR5 (green) and DAPI (blue). Scale bars, 50 µm. Staining repeated on two to three participants.

d RNAScope *in situ* hybridization of DEFA5 (red), LGR5 (green), and DAPI (blue) in bowel sections from the human duodenum. Scale bars, 10 µm. Staining repeated on two participants.

e Immunofluorescent (left) and immunohistochemical (middle) images showing the expression of DEFA5 in TA cells. Immunohistochemical (right, from https://proteinatlas.org) image showing the location of TA cells in the crypt by MKI67 staining. Scale bars, 50 µm in immunofluorescent image and 100µm in immunohistochemical images.

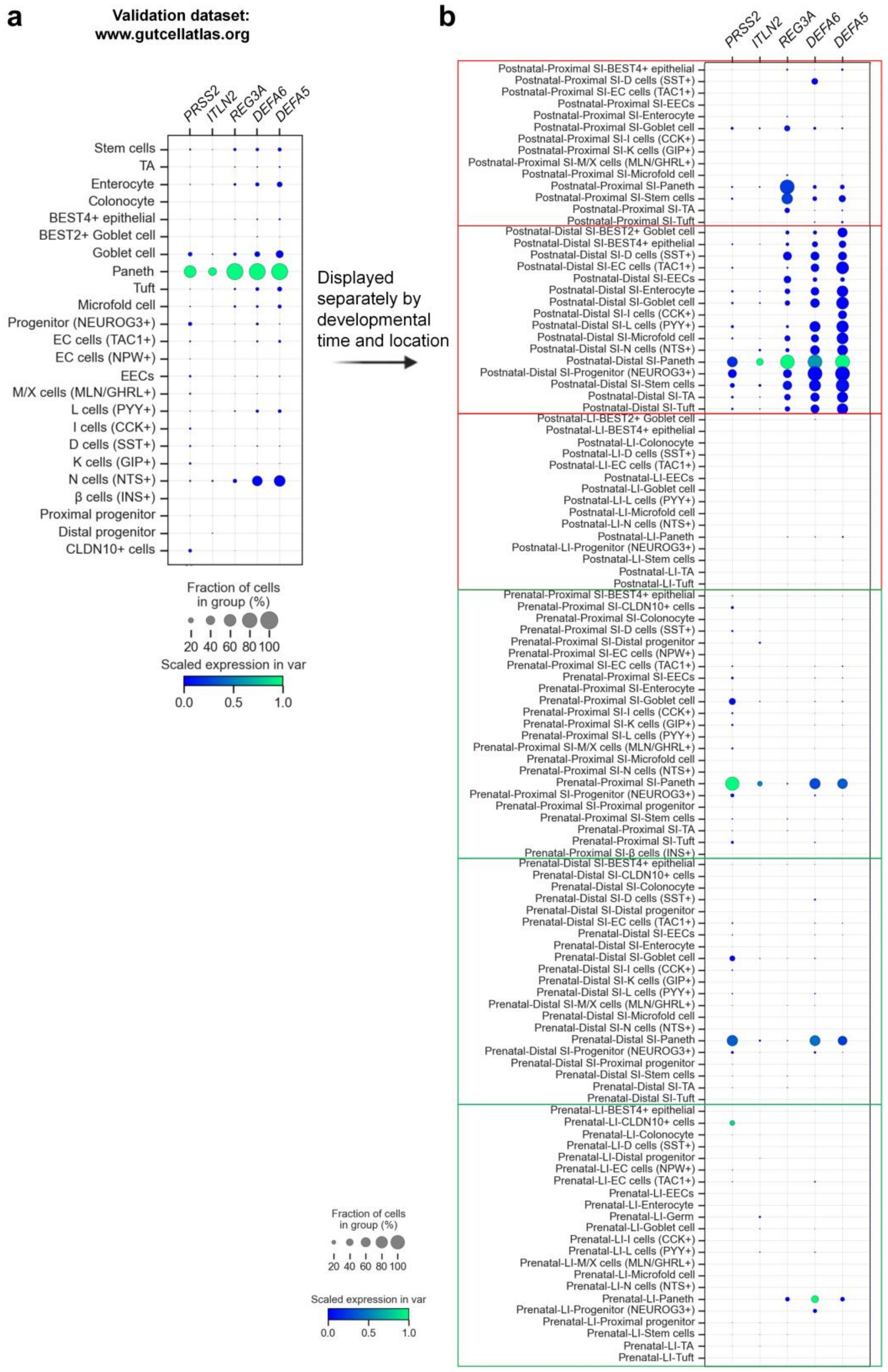

Supplemental Fig. 5 | SI-specific AMPs are ubiquitously expressed in human postnatal ileal epithelium.

a Dot plot showing the expression of SI-specific AMPs in epithelial cell lineages in the validation dataset (from www.gutcellatlas.org).

b Dot plot showing the expression of SI-specific AMPs in epithelial cell lineages grouped by developmental stage and anatomical location in the validation dataset (from www.gutcellatlas.org).

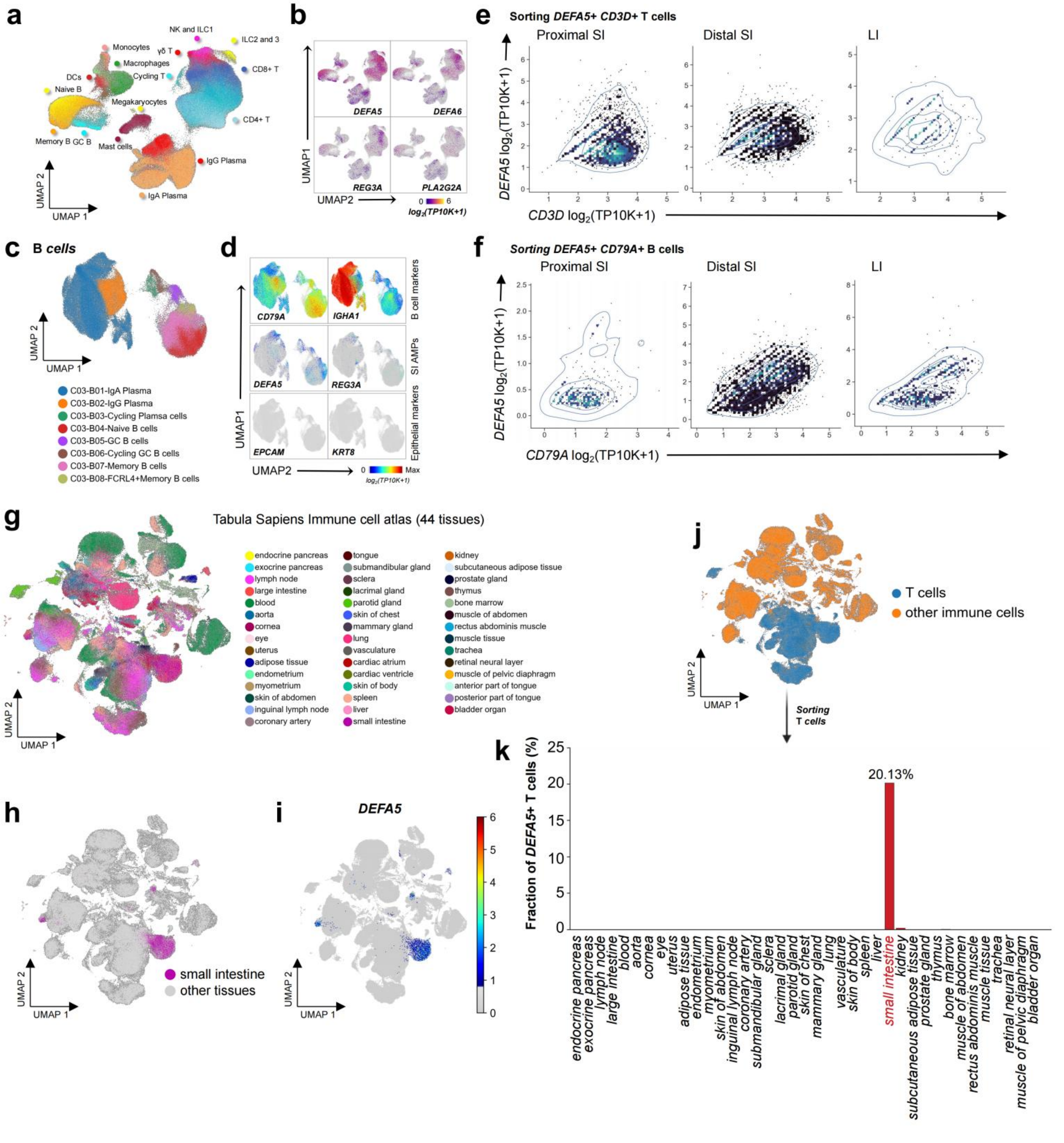

Supplemental Fig. 6 | SI immune cells also highly express SI-specific AMPs.

a UMAP embedding of human immune cells in scRNA-seq data.

b Feature plots showing the expression of SI-specific AMPs (*DEFA6, DEFA6, REG3A, REG3G*) in immune cell compartment.

c UMAP embedding of eight B/Plasma cell subsets.

d Feature plots showing the expression of B cell markers, SI-specific AMPs, and epithelial markers in B/Plasma compartment.

e, f Scatter plots showing the co-expression of representative SI-specific AMP (*DEFA5*) and the marker genes of T (e) and B cells (f).
g UMAP embedding of immune cells from 44 tissues (Tabula Sapiens cell atlas).
h, i UMAP embedding of immune cells coloured by anatomical location (small intestine, pink; other tissues, grey) (h), and representative SI-specific AMP (*DEFA5*) (i).
j UMAP embedding of immune cells coloured by cell types (T cells, blue; other immune cells, orange).
k Bar plot depicting the proportion of *DEFA5*+ cells in T cell compartment of 44 human tissues.

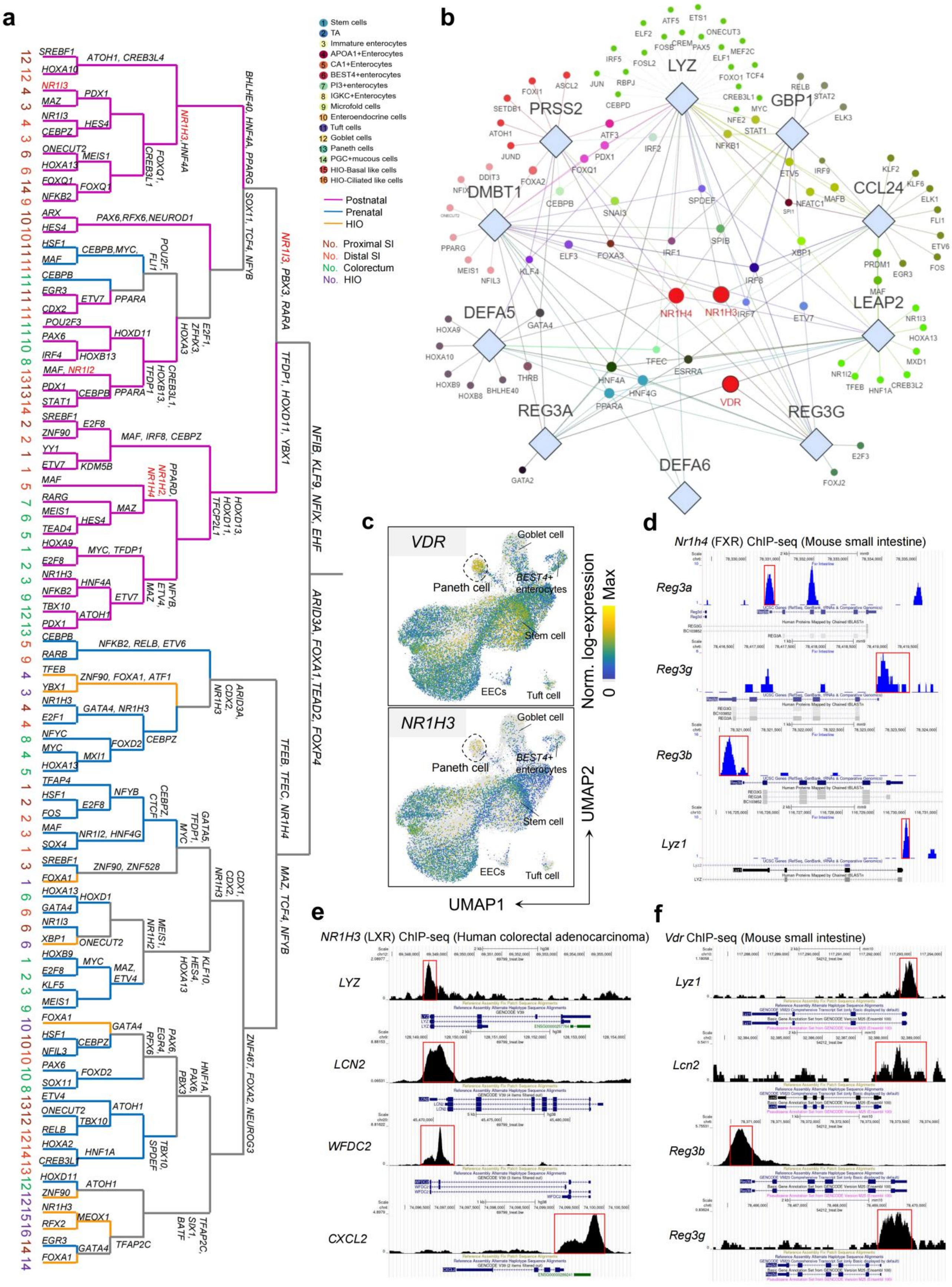

a
b
c
d
e
f
Stem cells
TA
Immature enterocytes
APOA1+Enterocytes
CA1+Enterocytes
BEST4+enterocytes
PI3+enterocytes
IGKC+Enterocytes
Microfold cells
Enteroendocrine cells
Tuft cells
Goblet cells
Paneth cells
PGC+mucous cells
HIO-Basal like cells
HIO-Ciliated like cells
Postnatal
Prenatal
HIO
No. Proximal SI
No. Distal SI
No. Colorectum
No. HIO
LYZ
PRSS2
GBP1
DMBT1
CCL24
DEFA5
LEAP2
REG3A
REG3G
DEFA6
NR1H4
NR1H3
VDR
Goblet cell
Paneth cell
BEST4+ enterocytes
Stem cell
EECs
Tuft cell
Norm. log-expression
Max
0
UMAP1
UMAP2
Nr1h4 (FXR) ChIP-seq (Mouse small intestine)
Reg3a
Reg3g
Reg3b
Lyz1
NR1H3 (LXR) ChIP-seq (Human colorectal adenocarcinoma)
LYZ
LCN2
WFDC2
CXCL2
Vdr ChIP-seq (Mouse small intestine)
Lyz1
Lcn2
Reg3b
Reg3g

Supplemental Fig. 7 | BATFs are the upstream regulators of intestinal AMPs.
a A dendrogram of regulons for epithelial cell subsets in different developmental stage and anatomical location. TFs at each branching point are representative regulons of subjacent groups. BATFs in postnatal SI lineages are highlighted. Branches are colored by development stage. Numbers represent the cell types and are colored by intestinal region.
b Venn network plot of SI-specific AMPs and their TFs highlighting the common upstream elements such as *NR1H3*, *NR1H4* and *VDR*.
c Feature plots showing mRNA expression of *VDR* and *NR1H3* in small intestinal epithelial cells.
d-f Small or large intestinal ChIP-seq analysis of FXR (d), LXR (e), and VDR (f) binding sites in AMP genes.

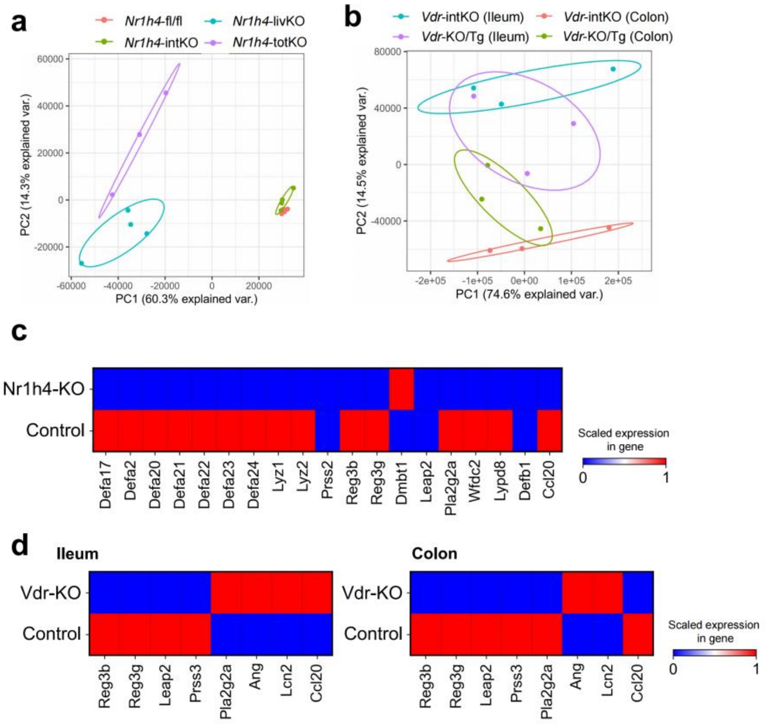

Supplemental Fig. 8 | Effects of perturbing BATFs *in vivo* on predicted targeted AMPs.

a, b PCA embedding demonstrating transcriptome of mice intestinal tissues with different genotypes (a, *Nr1h4* disturbance; b, *Vdr* disturbance).

c Heat map showing the expression of potential target genes of NR1H4 in *Nr1h4*-fl/fl (control) and *Nr1h4*-intKO mice.

d Heat map showing the expression of potential target genes of VDR in *Vdr*-intKO/TG (control) and *Vdr*-intKO mice.

Source data underlying **c** and **d** are provided as a Source Data file.

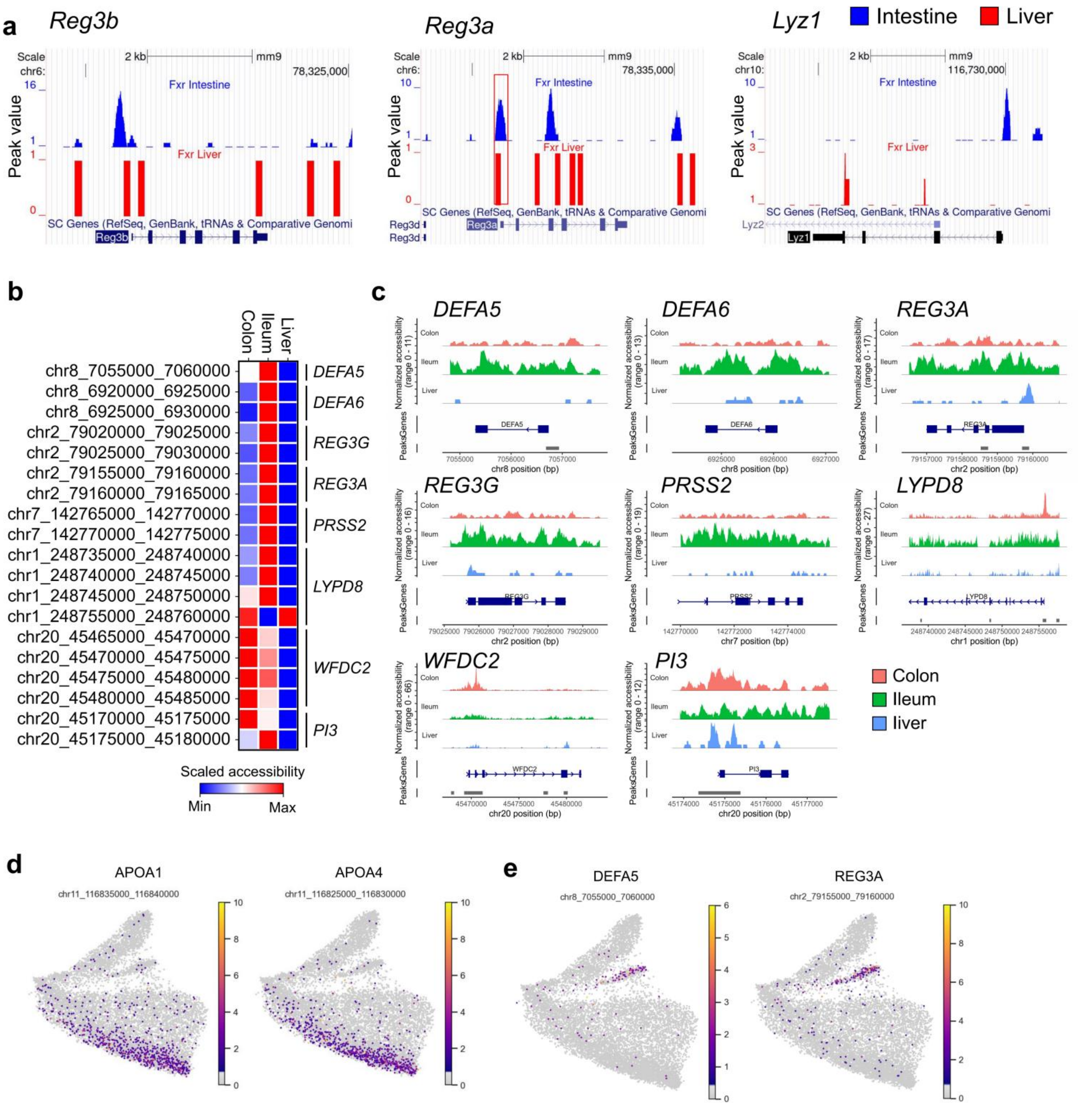

Supplemental Fig. 9 | Chromatin accessibility is involved in shaping the regional heterogeneity of AMPs.

a Intestinal (top) and hepatic (bottom) ChIP-seq analysis of FXR binding sites in *Reg3b*, *Reg3a*, and *Lyz1*.

b Chromatin accessibility of AMP-associated gene fragments in randomly sampled epithelial cells (n = 2500 cells, respectively) from three organs. The mean accessibility per organ is indicated with a colour scale from 0 (closed) to 1 (open).

c Aggregated single-cell profiles showed significant differences in chromatin accessibility of SI- (i.e. *DEFA5*, *DEFA6*, *REG3A*, *REG3G*, *PRSS2*), co-expression, and LI- (i.e. *LYPD8*, *WFDC2*, *PI3*) specific AMPs in human liver, ileum, and colon tissue. Cells included in the analysis were randomly selected from the three organs (n = 2500 cells, respectively) to avoid the bias of differences in cell numbers.

d, e UMAP embedding showing the chromatin accessibility of enterocyte markers (d, *APOA1* and *APOA4*) and Paneth cell markers (e, *DEFA5* and *REG3A*) in the human ileum.

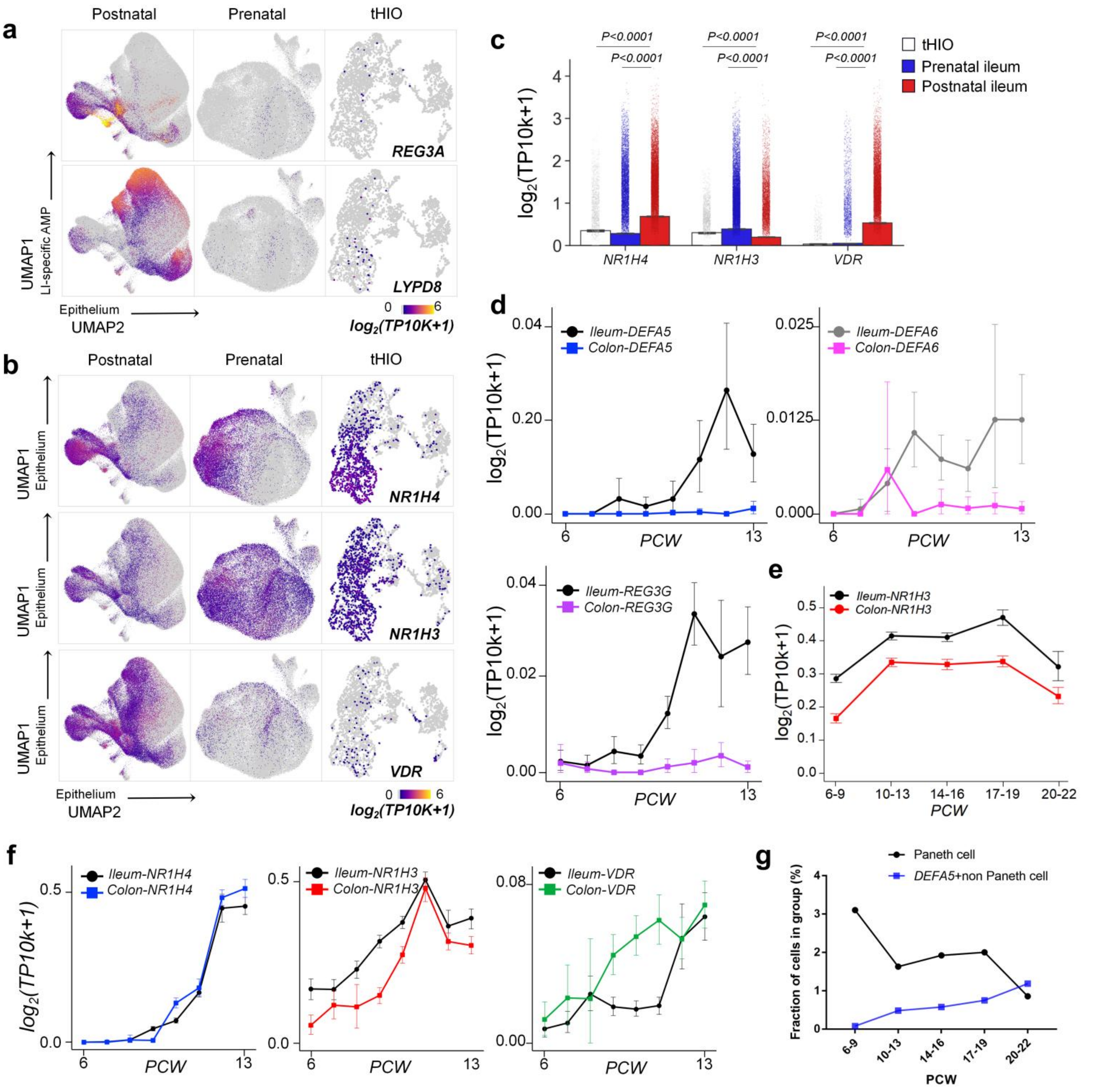

Supplemental Fig. 10 | BATFs-AMPs axis establishes intestinal AMPs immune surveillance before birth.

a Feature plots showing expression of selected AMP genes that varies in postnatal, prenatal and tHIO.

b, c Feature plots (b) and bar plots (c) showing expression of BATFs (*NR1H4*, *NR1H3*, and *VDR*) that varies in postnatal (n = 24094 cells), prenatal (n = 31916 cells) and tHIO (n = 3164 cells).

d Shown are changes in expression of SI-specific AMPs (*DEFA5*, *DEFA6* and *REG3G*) during 6-13 PCW of fetal development (n = 1055 and 715 cells for ileum and colon in 6 PCW respectively, n = 938 and 505 cells for ileum and colon in 7 PCW respectively, n = 1800 and 188 cells for ileum and colon in 8 PCW respectively, n = 4204 and 1978 cells for ileum and colon in 9 PCW respectively, n = 4991 and

2214 cells for ileum and colon in 10 PCW respectively, n = 2677 and 802 cells for ileum and colon in 11 PCW respectively, n = 1168 and 3148 cells for ileum and colon in 12 PCW respectively, n = 3020 and 2983 cells for ileum and colon in 13 PCW respectively).

e Regional heterogeneity of bile acid nuclear receptor (i.e. *NR1H3*) gradually established with fetal development (n = 7997 and 3386 cells for ileum and colon in 6-9 PCW respectively, n = 11856 and 9147 cells for ileum and colon in 10-13 PCW respectively, n = 8382 and 5510 cells for ileum and colon in 14-16 PCW respectively, n = 2746 and 4856 cells for ileum and colon in 17-19 PCW respectively, n = 935 and 1835 cells for ileum and colon in 20-22 PCW respectively).

f Shown are changes in expression of BATFs during 6-13 PCW of fetal development (n = 1055 and 715 cells for ileum and colon in 6 PCW respectively, n = 938 and 505 cells for ileum and colon in 7 PCW respectively, n = 1800 and 188 cells for ileum and colon in 8 PCW respectively, n = 4204 and 1978 cells for ileum and colon in 9 PCW respectively, n = 4991 and 2214 cells for ileum and colon in 10 PCW respectively, n = 2677 and 802 cells for ileum and colon in 11 PCW respectively, n = 1168 and 3148 cells for ileum and colon in 12 PCW respectively, n = 3020 and 2983 cells for ileum and colon in 13 PCW respectively).

g Shown are changes in the fraction of *DEFA5* positive non-Paneth cells and Paneth cells during 6-22 PCW of fetal development.

The length of the error bars is a 95% confidence interval for the mean in Supplemental Fig. 10. NS, not significant. All *p*-values were calculated and reported using two-tailed Student's *t*-test.

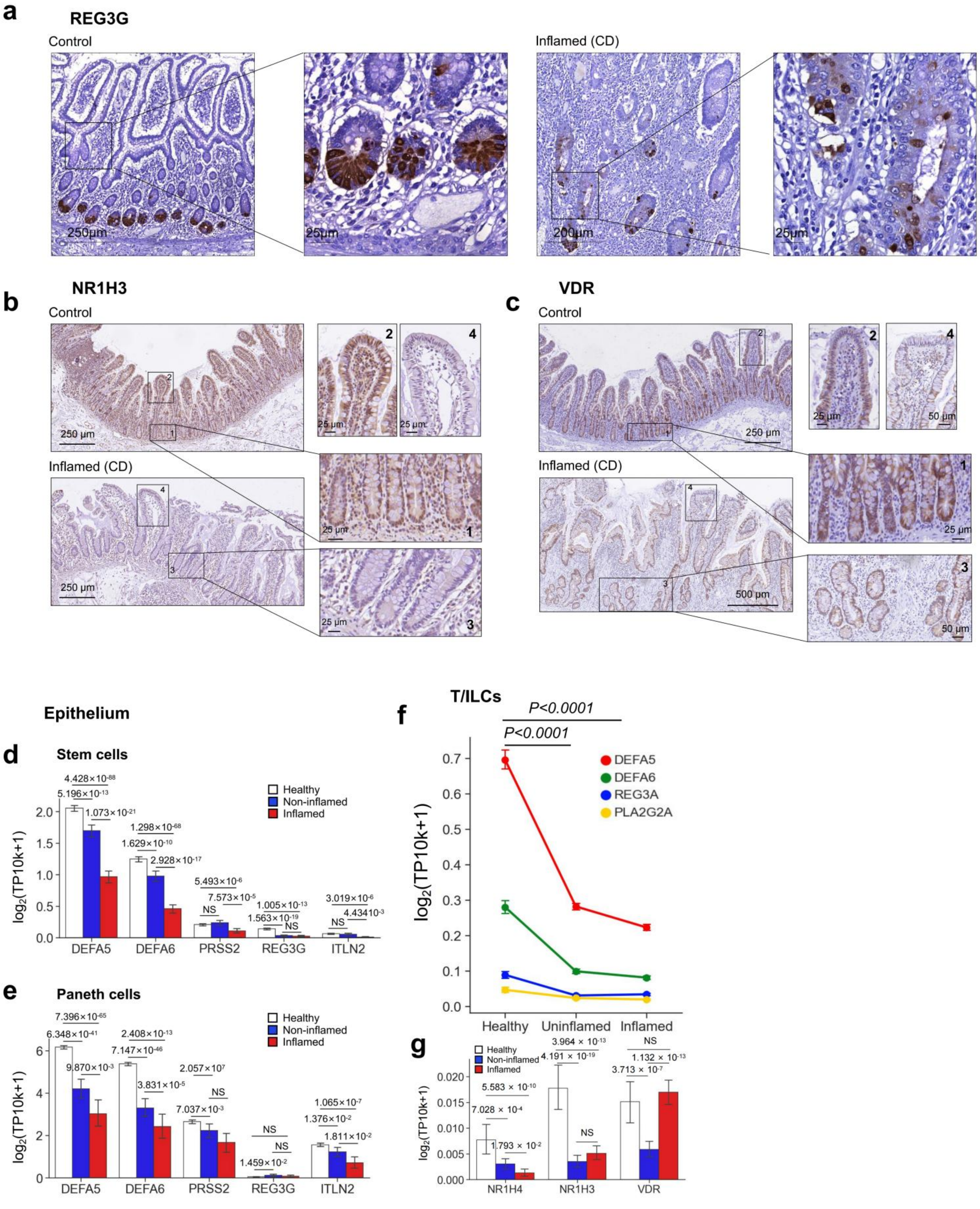

Supplemental Fig. 11 | Abnormal BATFs-AMPs axis mediates disruption of AMPs in Crohn's disease.

a Expression of REG3G in histological sections (n = 3 for each phenotype). Scale bars, 250 or 25 μm.
b, c Expression of NR1H3 (a) and VDR (b) in histological sections (n = 9 for each phenotype). Scale bars, 250, 50 or 25 μm.
d, e Shown are changes in expression of Paneth cell-AMPs (*DEFA5*, *DEFA6*, *PRSS2*, *REG3G*, *ITLN2*) in ileal stem cells (d) and Paneth cells (e) during CD progression (n = 3244, 1435, and 817 cells for stem cells in healthy, non-inflamed, and inflamed states respectively; n = 1958, 222, and 122 cells for Paneth cells in healthy, non-inflamed, and inflamed states respectively).
f, g Shown are changes in expression of AMPs (*DEFA5*, *DEFA6*, *REG3A*, *PLA2G2A*) (f) and BATFs (*NR1H4*, *NR1H3*, *VDR*) (g) in ileal T/ILCs during the progression of CD (n = 7786, 27107, and 27479 cells for T/ILCs in healthy, non-inflamed, and inflamed states respectively).
The length of the error bars is a 95% confidence interval for the mean in Supplementary Fig. 11. NS, not significant. All *p*-values were calculated and reported using two-tailed Student's *t*-test.
