# Supplementary material for: Bile acid-dependent transcription factors and chromatin accessibility determine regional heterogeneity of intestinal antimicrobial peptides
Source: Nat Commun. 2023 Aug 22;14:5093. doi: 10.1038/s41467-023-40565-7 (PMC10444805; doi:10.1038/s41467-023-40565-7)
Supplement: Supplementary file 3 — Description of Additional Supplementary Files [file 41467_2023_40565_MOESM3_ESM.pdf]

Description of Additional Supplementary Files

Supplementary Data 1.
Excel file containing participants details.

Supplementary Data 2.
Excel file containing key transcription factors at each branching point representing subjacent groups of regulons.

Supplementary Data 3.
Excel file containing the predicted upstream regulators depicted by ingenuity pathway analysis of genes enriched in absorptive enterocytes in a specific region versus absorptive enterocytes in the other two regions. The p-value indicates the statistical significance of the overlap between the analysis-ready genes and the downstream genes targeted by the upstream regulators (as calculated by the right-tailed Fisher's exact test)

Supplementary Data 4.
Excel file containing detailed information on the published RNA-seq and microarray datasets for bile acid-dependent nuclear receptor stimulation or knockout used in this paper.
